# Supersaturation of Dissolved Hydrogen and Methane in Rumen of Tibetan Sheep

**DOI:** 10.3389/fmicb.2016.00850

**Published:** 2016-06-14

**Authors:** Min Wang, Emilio M. Ungerfeld, Rong Wang, Chuan She Zhou, Zhu Zha Basang, Si Man Ao, Zhi Liang Tan

**Affiliations:** ^1^Key Laboratory for Agro-Ecological Processes in Subtropical Region, South Central Experimental Station of Animal Nutrition and Feed Science, Ministry of Agriculture, Institute of Subtropical Agriculture, The Chinese Academy of SciencesChangsha, China; ^2^Hunan Co-Innovation Center of Animal Production SafetyChangsha, China; ^3^Graduate University of Chinese Academy of SciencesBeijing, China; ^4^Instituto de Investigaciones Agropecuarias CarillancaTemuco, Chile; ^5^Department of Animal Science and Veterinary Medicine, Tibetan Autonomous Prefecture Academy of Agricultural and Animal Husbandry ScienceLhasa, China

**Keywords:** dissolved hydrogen, dissolved methane, equilibrium, rumen, fermentation pathways, volatile fatty acids

## Abstract

Hydrogen (H_2_) is an essential substrate for methanogens to produce methane (CH_4_), and also influences pathways of volatile fatty acids (VFA) production in the rumen. Dissolved H_2_ (H_2 (aq)_) is the form of H_2_ available to microbes, and dissolved CH_4_ (CH_4 (aq)_) is important for indicating methanogens activity. Rumen H_2 (aq)_ concentration has been estimated by assuming equilibrium with headspace gaseous H_2_ (H_2 (g)_) concentration using Henry's law, and has also been directly measured in the liquid phase in some *in vitro* and *in vivo* experiments. In this *in vivo* study, H_2 (aq)_ and CH_4 (aq)_ concentration measured directly in rumen fluid and their corresponding concentrations estimated from their gaseous phase concentrations, were compared to investigate the existence of equilibrium between the gas and liquid phases. Twenty-four Tibetan sheep were randomly assigned to two mixed diets containing the same concentrate mixed with oat grass (OG diet) or barley straw (BS diet). Rumen gaseous phase and contents were sampled using rumenocentesis and oral stomach tubing, respectively. Rumen H_2 (aq)_ and CH_4 (aq)_ concentration and VFA profile differed between sheep fed OG and BS diets. Measured H_2 (aq)_ and CH_4 (aq)_ concentration were greater than H_2 (aq)_ and CH_4 (aq)_ concentrations estimated using gas concentrations, indicating lack of equilibrium between gas and liquid phase and supersaturation of H_2_ and CH_4_ in rumen fluid. As a consequence, Gibbs energy changes (Δ*G*) estimated for various metabolic pathways were different when calculated using dissolved gases concentrations directly measured and when using dissolved gases concentrations assuming equilibrium with the gaseous phase. Dissolved CH_4_, but not CH_4 (g)_, was positively correlated with H_2 (aq)_. Both H_2 (aq)_ and H_2 (g)_ concentrations were positively correlated with the molar percentage of butyrate and negatively correlated with the molar percentage of acetate. In summary, rumen fluid was supersaturated with both H_2_ and CH_4_, and H_2 (aq)_ was closely associated with the VFA profile and CH_4 (aq)_ concentration. The assumption of equilibrium between dissolved gases and gaseous phase affected Δ*G* estimation.

## Introduction

Carbohydrates are mainly degraded through glycolysis to phosphoenolpyruvate and pyruvate in the rumen. Glycolysis and pyruvate oxidative decarboxylation to acetyl-CoA result in the release of reducing equivalents, which can eventually be transferred to protons, forming hydrogen (H_2_). Hydrogen must be removed to facilitate rumen fermentation of feed to produce volatile fatty acids (VFA) (Russell and Wallace, 1997; McAllister and Newbold, 2008). Methane (CH_4_) production by methanogenic archaea is the main electron sink in the rumen. However, the production of propionate and butyrate competes with methanogenesis for reducing equivalents, as the metabolism of glucose to propionate and to butyrate incorporates reducing equivalents or results in less reducing equivalents released per mol of hexose fermented compared to acetate production, respectively (Ellis et al., 2008; Janssen, 2010). Furthermore, Gibbs energy changes (Δ*G*) of CH_4_ and VFA production are largely controlled by H_2_ concentration (Janssen, 2010).

Concentration of dissolved H_2_ (H_2 (aq)_) in the rumen is central to the thermodynamics of H_2_-producing and H_2_-utilizing reactions (Janssen, 2010). Hackmann (2013) reported that for several ecosystems there were large differences between Δ*G* of biochemical pathways calculated using dissolved gases concentrations directly measured and using dissolved gases concentration estimated by assuming equilibrium between the liquid and gas phases. In a recent meta-analysis, the assumption of equilibrium between H_2 (aq)_ and H_2 (g)_ likely underestimated the thermodynamic feasibility of H_2_-incorporating pathways of *in vitro* rumen fermentation (Ungerfeld, 2015a). Dissolved H_2_ concentration has been directly measured in a few experiments with rumen *in vitro* cultures (Hungate, 1967; Wang et al., 2014) and in the rumen *in vivo* (Robinson et al., 1981; Wang et al., 2016). It would be important to compare in the same experiment H_2 (aq)_ concentration directly measured in the rumen with its estimation assuming equilibrium between H_2 (aq)_ and H_2 (g)_, to understand how the assumption of equilibrium between H_2 (aq)_ and H_2 (g)_ could affect the estimation of Δ*G* in the rumen.

Hydrogen is produced as H_2 (aq)_ and then evolves to the H_2 (g)_ pool (Wang et al., 2013). The hypothesis for the present study was that rumen fluid is supersaturated in H_2_ and CH_4_ with respect to the gas phase of the rumen. Based on Hackmann (2013), we define supersaturation as a physical stage at which the concentration of a dissolved gas is above the expected concentration that would result from equilibrium with its gaseous phase. We investigated the relationship between gaseous and dissolved H_2_ and CH_4_ in the rumen of growing Tibetan sheep fed two different diets. Implications with regard to estimated Δ*G* of different rumen pathways is analyzed and discussed.

## Materials and methods

### Animal and diets

This study was carried out in accordance with the recommendations of regulations of the Administration of Affairs Concerning Experimental Animals, the State Science and Technology Commission of P. R. China. The protocol was approved by the Laboratory Animal Ethical Commission of the Institute of Subtropical Agriculture, Chinese Academy of Sciences. The experiment was conducted at the research farm of the Academy of Agricultural and Animal Husbandry Sciences in Lhasa, Tibet, China (altitude = 3658 m, latitude = N29°30′, longitude = E91°15′, atmospheric pressure = 0.64 atm).

Twenty-four growing Tibetan sheep (body weight = 15.9 ± 1.92 kg) were blocked into two equal groups of males (*n* = 12) and females (*n* = 12). Sheep within each group were randomly and equally assigned to two experimental diets. The oat grass (OG) diet was formulated to meet the 1.3 times of maintenance metabolizable energy and crude protein requirements of sheep according to Zhang and Zhang (1998), and contained a pelleted concentrate mixed as a total mixed ration with oat grass at a 50:50 ratio (DM basis). The pelleted concentrate ingredients were (DM basis): 45 g/kg soybean meal, 470 g/kg corn, 424 g/kg wheat bran, 7 g/kg calcium carbonate, 9 g/kg palm oil, 9 g/kg sodium chloride and 36 g/kg minerals, and vitamins premix. The chemical composition of OG diet is shown in Table 1. The barley straw (BS) diet had the same pelleted concentrate of OG diet, and was formulated using barley straw to replace oat grass in the OG diet. The amount of BS diet allocated daily was set to be the same as that of OG diet. Both two diets were offered in two meals in equal proportions at 0900 and 1800. All sheep had free access to fresh water. The experiment comprised 30 d of adaptation to diets followed by a 2-days collection period. Feed refusals were recorded, and feed and refusals samples were collected, during the last 5 days (from days 26 to 30) of the adaption period.

**Table 1:** **Chemical composition of diets and dry matter intake of Tibetan sheep**.

**DM**	**Forage**		**Diet^a^**		**SEM**	***P*-value**
	**Oat grass**	**Barley straw**	**Oat grass**	**Barley straw**		
	**949**	**955**	**955**	**967**	**–**	**–**
**CHEMICAL COMPOSITION (g/kg DM)**						
OM	952	923	932	918	–	–
CP	43.4	14.2	73.9	59.4	–	–
NDF	597	709	483	539	–	–
ADF	387	458	264	300	–	–
Starch	75.0	61.0	186	179	–	–
EE	62.0	61.0	69.4	65.7	–	–
NFC	250	139	306	254	–	–
ME^b^ (MJ/kg DM)	1.41	1.11	7.76	7.13	–	–
DM intake (g/d)	–	–	609	582	13.3	0.32

*DM, dry matter; OM, organic matter; CP, crude protein; NDF, neutral detergent fiber; ADF, acid detergent fiber; EE, ether extract; NFE, non-fibrous carbohydrates, calculated by the equation of OM-CP-EE-CF*.

a *Forage plus concentrate diet (1:1). The concentrate contained (g/kg) soybean meal (45), corn (470), wheat bran (424), calcium carbonate (7), palm oil (9), sodium chloride (9), and premix (36)*.

b *Metabolizable energy (ME) was estimated according to Zhang and Zhang (1998)*.

The chemical composition of forage and total mixed ration is provided in Table 1. Contents of organic matter, gross energy and Kjeldahl N (CP = 6.25 × N) were determined according to AOAC (1995). Neutral detergent fiber (NDF) and acid detergent fiber were expressed inclusive of residual ash (Van Soest et al., 1991), and NDF assayed with the addition of a heat stable amylase, but without sodium sulfite. Starch content was determined after pre-extraction with ethanol (80%), and glucose released from starch by enzyme hydrolysis was measured using amyloglucosidase (Kartchner and Theurer, 1981).

### Rumen sampling

Samples of rumen gas and contents were collected before the morning feeding in the last 2 days of the collection period, with 12 sheep in each day (six males and six females randomly chosen within each diet). Rumen headspace gas was sampled using the rumenocentesis method of Moate et al. (1997) with a slight modification. A 150-mm, 14-g needle was inserted into the headspace of the rumen via the central area of the left paralumbar fossa, which had been trimmed of hair and then swabbed with 72% ethanol. A 50-mL syringe, fitted with a T-shaped tube, was attached to the 150-mm needle to collect 30 mL of headspace gas from the rumen. The collected gas was then injected into 10-mL evacuated tubes for subsequent determination of H_2 (g)_ and CH_4 (g)_ concentration.

Rumen contents were sampled using oral stomach tubing immediately after collecting rumen headspace gas. A flexible PVC tube (2 mm of wall thickness and 6 mm of internal diameter) was warmed-up using hot water (about 50°C) and inserted to a depth of ~120–150 cm via the esophagus. The first 100-mL of rumen contents were discarded to avoid saliva contamination, and the following 150 mL of rumen contents were rapidly collected for subsequent determination of rumen H_2 (aq)_ and CH_4 (aq)_ concentrations and fermentation end-products. Two 35-mL subsamples were immediately transferred to 50-mL plastic syringes for measuring H_2 (aq)_ and CH_4 (aq)_ concentration as explained below (see section Measured Dissolved Gases Concentration). The rumen pH was measured immediately after sampling using a portable pH meter (Starter 300; Ohaus Instruments Co. Ltd., Shanghai, China).

### Analyses of fermentation end products

Two milliliters samples of strained rumen fluid were centrifuged at 15,000 g for 10 min at 4°C. One and a half milliliters of supernatant were transferred into tubes containing 0.15 mL of 25% (w/v) metaphosphoric acid. The mixture was vigorously hand-shaken and stored at −20°C for subsequent determination of fermentation end-products. After thawing, the acidified samples were re-centrifuged at 15,000 g for 10 min at 4°C, the pellet discarded, and volatile fatty acids (VFA) analyzed in the supernatant using gas chromatography (Agilent 7890A, Agilent Inc., Palo Alto, CA), according to the method described by Wang et al. (2014). Ammonia, lactic acid, and glucose were determined colorimetrically according to the methods of Weatherburn (1967), Taylor (1996), and Nelson (1944) respectively.

### Determination of headspace gases concentration

Concentration of H_2 (g)_ and CH_4 (g)_ in the collected rumen headspace gas were determined by gas chromatography (Agilent 7890A, Agilent Inc., Palo Alto, CA) using a thermal conductivity and a flame ionization detector, respectively. Hydrogen and CH_4_ were separated using a Hayesep Q packed column (2.44 m × 1/8 in. × 2.0 mm ID). Carbon dioxide concentration in the rumen headspace gas was calculated as the difference between total gas concentration at the local atmospheric pressure, calculated in turn using the Ideal Gas Law (0.0262 M at 0.64 atm), and the sum of H_2 (g)_ and CH_4 (g)_ concentrations.

### Estimation of dissolved gases concentration based on headspace gases concentration

Concentrations of H_2 (aq)_ and CH_4 (aq)_ estimated as if they were at equilibrium with the gaseous phase were calculated based on rumen headspace H_2 (g)_ and CH_4 (g)_ concentrations, respectively. Wiesenburg and Guinasso (1979) proposed calculating gas solubility based on the Bunsen absorption coefficient, vapor pressure, gas concentration, atmospheric pressure, and relative humidity, according to:

(1) $$Gas{\;}_{\left(aq\right)}=\alpha Gas{\;}_{\left(g\right)}(P_{t}-P_{VP}h/100)$$

where *Gas*_(*aq*)_ is the concentration of the dissolved gas of interest in the liquid phase (mM), α is the Bunsen absorption coefficient of the gas of interest (L of dissolved gas/(L of liquid·atm), *Gas*_(*g*)_ is the concentration of the gas of interest in the gas phase (mM), *P*_*t*_ is atmospheric pressure (atm), *h* is the relative humidity (%), and *P*_*vp*_ is the liquid vapor pressure (atm).

The vapor pressure is greatly affected by the concentration of dissolved salts (Wiesenburg and Guinasso, 1979). Our previous study indicated that Bunsen absorption coefficients were similar for pure water and the McDougall's buffer at the same temperature (Wang et al., 2014), therefore the factor including vapor pressure in the McDougall's buffer could be assumed to be the same as pure water, and then set to be zero in Equation (1) to calculate the dissolved gas in rumen fluid. Concentrations of H_2 (aq)_ and CH_4 (aq)_ estimated from H_2 (g)_ and CH_4 (g)_ concentrations (eH_2 (aq)_ and eCH_4 (aq)_, respectively), in the rumen headspace were calculated as:

(2a) $$eGas{\;}_{\left(aq\right)}=Gas{\;}_{\left(g\right)}\alpha_{gas}P_{t}$$

with:

(2b) $$\begin{array}{l} \alpha_{H2}=\exp(-47.8948+65.0368(100/T)\\ \;+\;20.1709\ln\;(T/100)) \end{array}$$

(2c) $$\begin{array}{l} \alpha_{CH4}=\exp(-68.8862+101.4953(100/T)\\ \;+\;28.7314\ln\;(T/100)) \end{array}$$

where *eGas*_(*aq*)_ is the estimated concentration of the dissolved gas of interest (mM), *Gas*_(*g*)_ is the corresponding gas concentration measured in the rumen headspace (mM), α_*gas*_ is the Bunsen absorption coefficient for each gas of interest (H_2_ or CH_4_) at 1 atm pressure (L of dissolved gas/(L of liquid·atm) calculated as function of absolute temperature *T* for a rumen temperature of 39°C (Wiesenburg and Guinasso, 1979), and *P*_*t*_ is 0.64 atm.

### Measured dissolved gases concentration

Rumen H_2 (aq)_ and CH_4 (aq)_ concentration were measured by establishing an equilibrium between the gas and the liquid phase in a sealed vessel containing a rumen fluid sample using the procedure decribed by Wang et al. (2014). Briefly, a 50-mL plastic syringe, containing 35 mL rumen fluid, was fitted with a T tube, which was closed immediately and cooled to room temperature. A 20-mL syringe was filled with 10 mL of N_2_ gas, and connected to 50-mL plastic syringe via the T tube. The N_2_ gas was then injected into the 50-mL syringe through the T tube, and the gases dissolved in the rumen fluid were extracted into the N_2_ gas phase by vigorous hand shaking for 5 min. The volumes of gas and liquid phases were recorded using the scales in the small and large syringes, respectively. The room temperature was recorded as well. Gas samples from the 20-mL syringe were collected in evacuated tubes for the measurement of H_2_ and CH_4_ concentration using gas chromatography as above described (Agilent 7890A, Agilent Inc., Palo Alto, CA, USA).

Total H_2_ or CH_4_ before extraction is equal to that after extraction:

(3) $$V_{l}Gas{\;}_{\left(aq\right)}=V_{l}Gas{\;}_{\left(g\right)}\alpha {\;}_{gas}P_{t}+V{\;}_{g}Gas{\;}_{\left(g\right)}$$

where *V*_l_ is the liquid volume (mL); *Gas*_(*aq*)_ is the concentration of the dissolved target gas (H_2_ or CH_4_) in the original liquid sample (mM), *Gas*_(*g*)_ is the target gas (H_2_ or CH_4_) concentration in the gas phase at equilibrium after extraction (mM), α_*gas*_ is the Bunsen absorption coefficient of each target gas (H_2_ or CH_4_) at room temperature, as calculated using Equations (2b) and (2c) (L of dissolved gas/(L of liquid·atm), *P*_*t*_ is the local atmospheric pressure equal to 0.64 atm, and *V*_*g*_ is the gas volume at equilibrium after extraction (mL).

Therefore, the rumen aqueous concentration of each dissolved target gas (H_2_ or CH_4_) is equal to:

(4) $$Gas{\;}_{\left(aq\right)}=Gas{\;}_{\left(g\right)}(\alpha {\;}_{gas}P_{t}+V_{g}/V_{l})$$

We could not use the method described by Wang et al. (2014) to determine the concentration of dissolved CO_2_(CO_2 (aq)_), because the release of CO_2_ in solution toward the gas phase would displace the equilibrium from bicarbonate and carbonic acid toward additional CO_2 (aq)_ and finally extra CO_2 (g)_. For Δ*G* calculations, CO_2 (aq)_ concentration was calculated using Eq. 2 by assuming equilibrium with CO_2 (g)_ at the local atmospheric pressure of 0.64 atm. The Bunsen absorption coefficient for CO_2_ in rumen fluid was set to be 0.234 volume/(volume atm) (Hille et al., 2016).

### Calculation of the saturation factor

The saturation factor (*Sf*) was defined as the ratio between the measured dissolved gases (H_2 (aq)_ and CH_4 (aq)_) concentration, and the concentration of dissolved gases estimated based on headspace H_2 (g)_ and CH_4 (g)_ concentrations (eH_2 (aq)_ and eCH_4 (aq)_)(Wang et al., 2014):

(5) $$Sf_{gas}=\frac{Gas{\;}_{\left(aq\right)}}{eGas{\;}_{\left(aq\right)}}$$

“*Sf*_*gas*_ > 1” and “*Sf*_*gas*_ < 1” indicate supersaturation and undersaturation of the dissolved gas in the liquid phase of the rumen, respectively.

### Calculation of the gibbs energy changes of fermentation pathways

Ingested dietary polysaccharides are hydrolyzed to hexoses in the rumen, which are fermented to VFA. Acetate production results in the release of reducing equivalents:

(Reaction 1) $${\text{C}}_{6}{\text{H}}_{12}{\text{O}}_{6}+2\;{\text{H}}_{2}\text{O}\to 2\text{ acetate}+2\;{\text{CO}}_{2}+4\;[2\text{H}]+2\;{\text{H}}^{+}$$

Interconversion between VFA has been shown to occur in the rumen (Ungerfeld and Kohn, 2006). Conversion of acetate to propionate and butyrate incorporates reducing equivalents:

(Reaction 2) $$\text{acetate}+{\text{CO}}_{2}+3\;[2\text{H}]\to \text{propionate}+2\;{\text{H}}_{2}\text{O}$$

(Reaction 3) $$2\text{ acetate}+{\text{H}}^{+}+2\;[2\text{H}]\to \text{butyrate}+2\;{\text{H}}_{2}\text{O}$$

Fermentation shifts of acetate to propionate and acetate to butyrate then result in incorporation of reducing equivalents. Different examples of fermentation stoichiometries with differing [2H] release per mol of hexose fermented (Janssen, 2010), expressed as H_2_ production in the following equations, can be generated by replacing the products of Reaction 1 with the reactants of Reaction 2 and 3:

(Reaction 4) $$\begin{array}{l} {\text{C}}_{6}{\text{H}}_{12}{\text{O}}_{6}\to 2/3\text{ acetate}+4/3\text{ propionate}+2/3\;{\text{CO}}_{2}\\ \;+\;2/3\;{\text{H}}_{2}\text{O}+2{\text{H}}^{+} \end{array}$$

(Reaction 5) $${\text{C}}_{6}{\text{H}}_{12}{\text{O}}_{6}\to \text{acetate}+\text{propionate}+{\text{CO}}_{2}+{\text{H}}_{2}+\;2\;{\text{H}}^{+}$$

(Reaction 6) $${\text{C}}_{6}{\text{H}}_{12}{\text{O}}_{6}\to \text{butyrate}+{\text{H}}^{+}+2\;{\text{CO}}_{2}\;+2\;{\text{H}}_{2}$$

(Reaction 7) $$\begin{array}{l} {\text{C}}_{6}{\text{H}}_{12}{\text{O}}_{6}+{\text{H}}_{2}\text{O}\to \text{acetate}+1/2\text{ butyrate}+2\;{\text{CO}}_{2}+3\;{\text{H}}_{2}\\ \;+\;3/2\;{\text{H}}^{+} \end{array}$$

The amount H_2_ production per mol of hexose fermented can thus vary widely.

Another two pathways of H_2_ incorporation are reductive acetogenesis for acetate production and methanogenesis for CH_4_ production:

(Reaction 8) $$2\;{\text{CO}}_{2}+4\;{\text{H}}_{2}\to \text{acetate}+{\text{H}}^{+}+2\;{\text{H}}_{2}\text{O}$$

(Reaction 9) $${\text{CO}}_{2}+4{\text{H}}_{2}\to {\text{CH}}_{4}+2\;{\text{H}}_{2}\text{O}$$

The thermodynamic feasibility of these reactions was estimated through their Δ*G*. Gibbs energy changes of reactions at standard conditions (Δ*G*°) were calculated from standard Gibbs energy of formation (Δ*G*°_*f*_) of reactants and products (Kohn and Boston, 2000):

(6) $$\Delta G{}^{\circ}=\Delta G_{f\text{products}}^{0}-\Delta G_{f\text{reactants}}^{0}$$

Standard Gibbs energy changes so calculated for 298 K (0°C) were then adjusted to a rumen temperature of 312 K using the van't Hoff equation (Kohn and Boston, 2000). Gibbs energy changes estimated for actual rumen conditions were subsequently adjusted by the concentration of soluble metabolites and dissolved gases (Kohn and Boston, 2000):

(7) $$\begin{array}{l} \Delta G=\Delta G{}^{\circ}+\text{R}T\text{Ln}(\Pi_{\text{i }=\;1}{}^{\text{i }=\text{ n}}{\left[\text{Product}\right]}_{\text{i}}{}^{\text{product i}}/\\ \;\Pi_{\text{i }=\;1}{}^{\text{i }=\text{ n}}\;{\left[{\text{Reactant}}_{\text{i}}\right]}^{\text{reactant i}}) \end{array}$$

where $\Pi_{\text{i }=\;1}{}^{\text{i }=\text{ n}}$ [Products] and $\Pi_{\text{i }=\;1}^{\text{i }=\text{ n}}$ [Reactants] are the products of molar concentration of products and reactants in the liquid phase, respectively, each elevated to its corresponding stoichiometric coefficient, *R* is the gas constant equal to 8.314 J atm K^−1^ mol^−1^, *T* is the rumen temperature in Kelvin, and Δ*G*° is the standard Δ*G* for the reaction adjusted to 39°C. Reactions are thermodynamically feasible if Δ*G* < 0, at equilibrium if estimated Δ*G* = 0, and unfeasible when Δ*G* > 0.

Gibbs energy changes of various fermentation stoichiometries, as well as of methanogenesis, reductive acetogenesis, and VFA interconversions were calculated and compared using either directly measured CH_4 (aq)_ and H_2 (aq)_ concentrations, or concentrations of CH_4 (aq)_ and H_2 (aq)_ estimated from CH_4 (g)_ and H_2 (g)_ concentrations using Henry's Law by assuming equilibrium between gaseous and dissolved gases. Gibbs energy changes calculated using both methods shared the estimation of CO_2 (aq)_ and measured glucose and individual VFA concentration.

### Statistics

The effect of diet on DM intake, rumen pH, and the concentrations and supersaturation indexes of gases, total VFA concentration, individual VFA molar percentages and ammonia concentration were evaluated through a one-way ANOVA.

Concentrations of H_2 (g)_, CH_4 (aq)_, CH_4 (g)_, and VFA molar percentages, were regressed against H_2 (aq)_ concentration as follows:

$$\begin{array}{l} y=\text{intercept}+{\text{H}}_{2\;(\text{aq})}+{\text{H}}_{2(\text{aq})}^{2}+\text{diet}+{\text{H}}_{2\;(\text{aq})}\times \text{diet}\\ \;+\text{ residual} \end{array}$$

Statistical significance was set at *P* < 0.05 and tendencies at 0.05 ≤ *P* ≤ 0.10. The main effect of the diet, the quadratic effect of H_2 (aq)_ and the interaction were removed from the models if their *P* > 0.10, and the reduced models re-fitted.

Gibbs energy changes for nine different pathways were analyzed as 2 × 2 factorials including the main effects of diet, method of estimation (calculation using CH_4 (aq)_ and H_2 (aq)_ concentration directly measured or using eCH_4 (aq)_ and eH_2 (aq)_ concentration estimated from CH_4 (g)_ and H_2 (g)_ concentration), and their interaction.

All statistical analyses were conducted with JMP® 12.1.0. (SAS Institute Inc.).

## Results

Sheep on the OG and BS diets had similar DM intake (*P* = 0.32; Table 1), rumen pH (*P* = 0.20), total VFA concentration (*P* = 0.17), and propionate (*P* = 0.60) and valerate (*P* = 0.102) molar percentages (Table 2), as well as CO_2 (g)_ (*P* = 0.74) and CH_4 (g)_ (*P* = 0.76) concentrations, and *Sf*_*H*2_ (*P* = 0.67; Table 3). Rumen glucose (*P* = 0.002), H_2 (aq)_ (*P* = 0.017), CH_4 (aq)_ (*P* = 0.07), H_2 (g)_ (*P* = 0.001), butyrate molar percentage (*P* < 0.001), and *Sf*_*CH*4_ (*P* = 0.08), were greater or tended to be greater in sheep fed the OG diet. Acetate (*P* = 0.022), *iso*-butyrate (*P* = 0.053), and *iso*-valerate (*P* = 0.065), and ammonia concentration (*P* = 0.041) molar percentages were greater or trended to be greater in sheep fed the BS diet (Table 2).

**Table 2:** **Fermentation end-productions in the rumen of Tibetan sheep feeding by two diets (*n* = 12)**.

	**Diet**		**SEM**	***P*****-value**
	**Oat grass**	**Barley straw**		
pH	6.85	6.94	0.047	0.20
Total VFA (mM)	109	81.0	5.51	0.17
**INDIVIDUAL VFA MOLAR PERCENTAGE (%)**				
Acetate	66.6	69.8	0.90	0.021
Propionate	19.5	18.9	0.78	0.60
Butyrate	10.5	7.75	0.41	<0.001
Valerate	0.93	0.81	0.051	0.102
*Iso*-butyrate	1.40	1.62	0.077	0.053
*Iso*-valerate	0.97	1.12	0.055	0.065
Acetate:Propionate (mol/mol)	3.51	3.73	0.171	0.36
Lactate (mM)	1.30	1.25	0.14	0.81
Glucose (mM)	2.64	1.88	0.11	0.002
Ammonia (mM)	6.09	8.18	0.0678	0.041

*BS, barley straw; OG, oat grass; VFA, volatile fatty acids*.

**Table 3:** **Gaseous and dissolved gases concentration in the rumen of Tibetan sheep fed two different diets (*n* = 12)**.

	**Diet**		**SEM**	***P*****-value**
	**Oat grass**	**Barley straw**		
H_2 aq_ (μM)	6.49	2.34	1.13	0.017
CH_4 (aq)_ (mM)	0.932	0.680	0.0943	0.072
H_2 (g)_ (μM)	7.86	3.36	0.852	0.001
CH_4 (g)_ (mM)	4.15	4.11	0.111	0.76
CO_2 (g)_ (mM)	22.1	22.2	0.114	0.74
*Sf*_*H*2_	42.7^*^	38.4^*^	6.90	0.67
*Sf*_*CH*4_	7.88^*^	5.79^*^	0.81	0.08

*BS, barley straw; CH_4 (aq)_, dissolved methane concentration in the original liquid sample; CH_4 (g)_, gaseous methane concentration in the rumen; H_2 (aq)_, dissolved hydrogen concentration in the original liquid sample; H_2 (g)_, gaseous hydrogen concentration in the rumen; OG, oat grass; Sf_CH4_, CH_4_ saturation factor; Sf_H2_, H_2_ saturation factor*.

* *Saturation factor significantly (*P* < 0.05) different from unity*.

**Figure 1:**
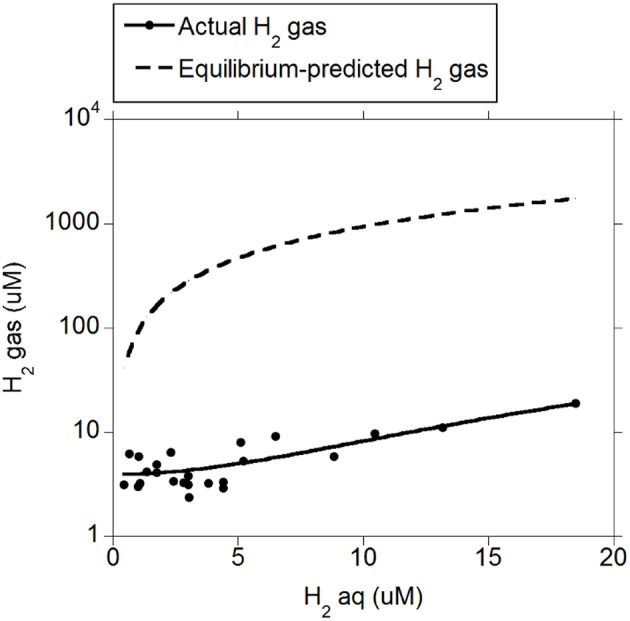
**Relationship between measured and equilibrium-predicted gaseous H_2_ and dissolved H_2_ concentration: H_2 (g)_ = 3.83 (±0.43; *P* < 0.001) ± 1.20 (±0.28; *P* < 0.001) diet + 0.18 (±0.11; *P* = 0.11) (H_2(aq)_) + 0.052 (±0.011; *P* < 0.001) (H_2 (aq)_ − 4.42)^2^; R^2^ = 0.91 (*P* < 0.001); Equilibrium-predicted H_2 (*g*)_: H_2 (aq)_/(α × *P*_*t*_), where α is the Bunsen coefficient and *P*_*t*_ is the atmospheric pressure**.

**Figure 2:**
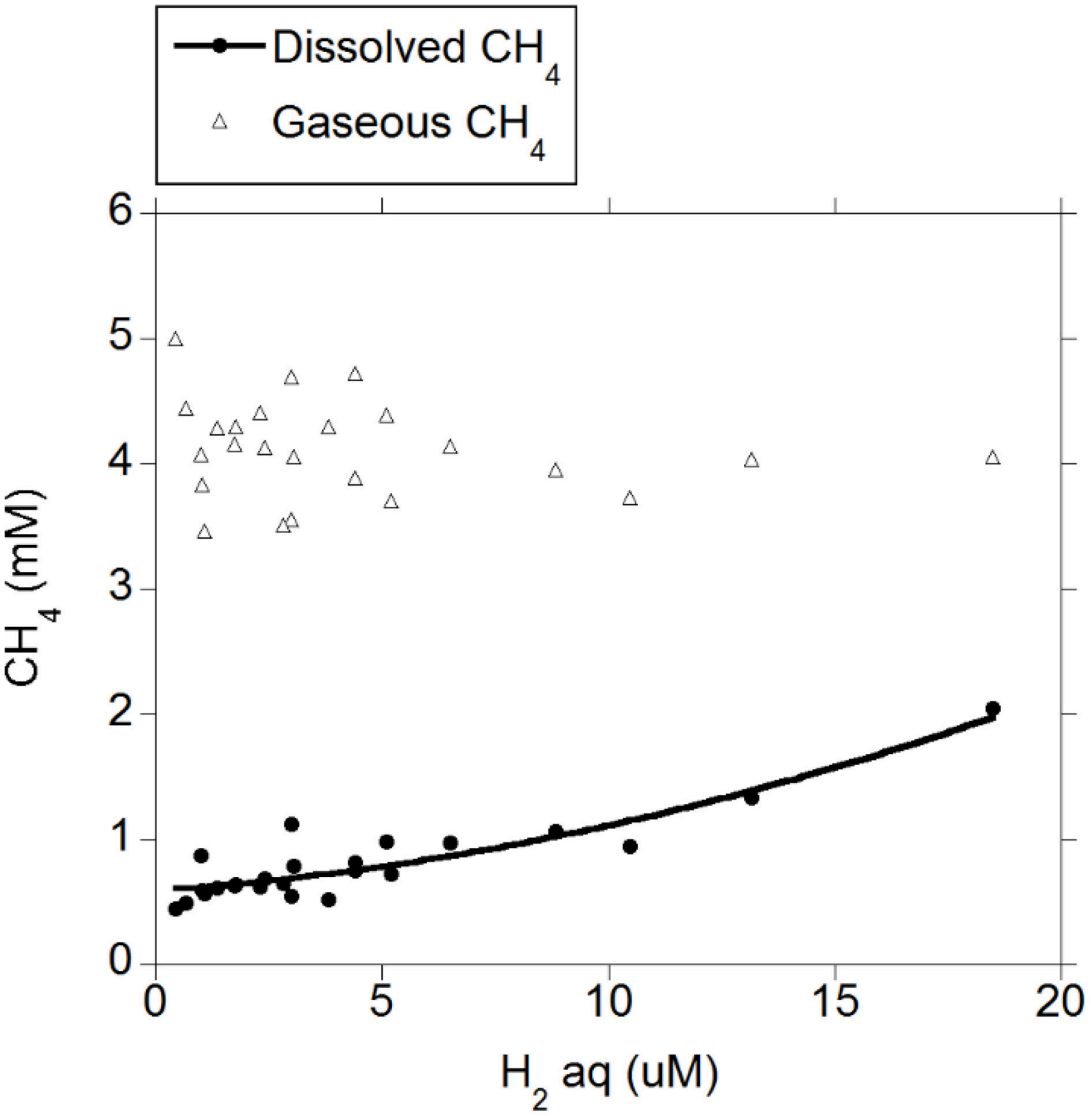
**Relationship of dissolved H_2_ concentration with dissolved and gaseous CH_4_: CH_4 (*aq*)_ = 0.54 (±0.049; *P* < 0.001) + 0.049 (±0.013; *P* < 0.001) H_2 (aq)_ + 0.0027 (±0.0013; *P* = 0.056) (H_2 (aq)_ – 4.42)^2^; *R*^2^ = 0.82 (*P* < 0.001); Gaseous CH_4_: CH_4 (g)_ = 4.20 (±0.11; *P* < 0.001) − 0.016 (±0.019; *P* = 0.40) H_2 (aq)_; *R*^2^ = 0.032 (*P* = 0.40)**.

**Figure 3:**
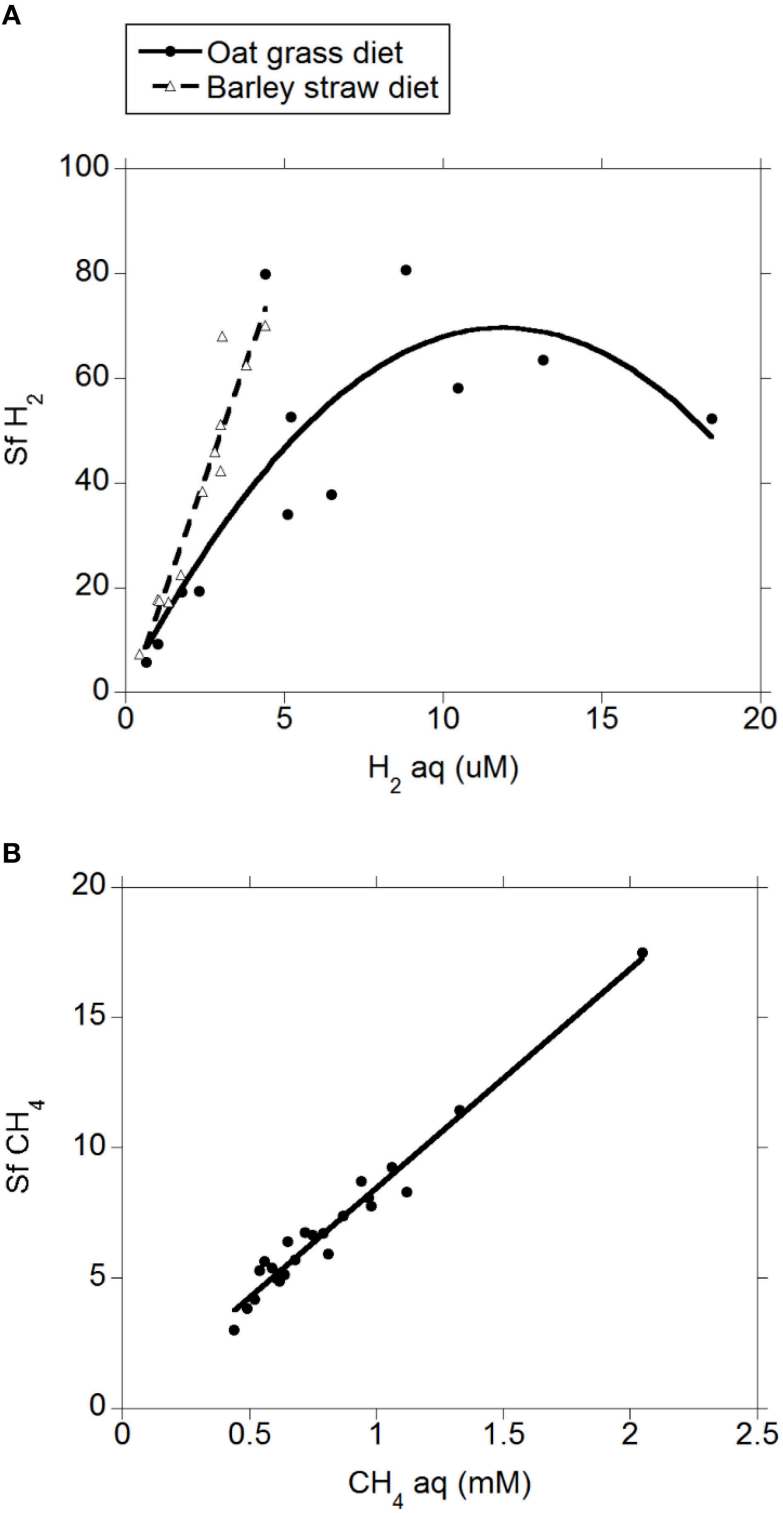
**Relationship between saturation factor and dissolved gases concentration. (A)** *Sf* H_2_ = 8.29 (±4.89; *P* = 0.11) ± 14.9 (±4.16; *P* = 0.002) diet + 11.2 (±1.57; *P* < 0.001) H_2_ (aq) − 0.48 (±0.12; *P* < 0.001) (H_2 (aq)_ − 4.42)^2^ ± 3.936 (H_2_ (aq) − 4.42) × diet; R^2^ = 0.78 (*P* < 0.001); **(B)** *Sf* CH_4_ = 0.048 (±0.30; *P* = 0.87) + 8.42 (±0.34; *P* < 0.001) CH_4 (aq)_; R^2^ = 0.97; *P* < 0.001).

There was a positive quadratic relationship between H_2 (g)_ and H_2 (aq)_ (*R*^2^ = 0.91; *P* < 0.001; Figure 1) without an interaction with the diet (*P* = 0.90). Measured H_2 (g)_ was roughly between one and two orders of magnitude smaller than H_2 (g)_ concentration predicted by assuming equilibrium with H_2 (aq)_ (Figure 1). There was a positive relationship between CH_4 (aq)_ and H_2 (aq)_ (*P* < 0.001) with a tendency to be quadratic (*P* = 0.056; Figure 2) and without an interaction with diet (*P* = 0.95). However, there was no relationship between CH_4 (g)_ and H_2 (aq)_ (*P* = 0.40; Figure 2), and no relationship between CH_4 (g)_ and CH_4 (aq)_ (*P* = 0.91; not shown). The saturation factors for H_2_ and CH_4_ were greater than unity (*P* < 0.001; Table 3) and positively correlated to dissolved gases concentrations (Figures 3A,B). The response of H_2_ saturation factor to H_2 (aq)_ was affected by the diet (Figure 3A).

**Figure 4:**
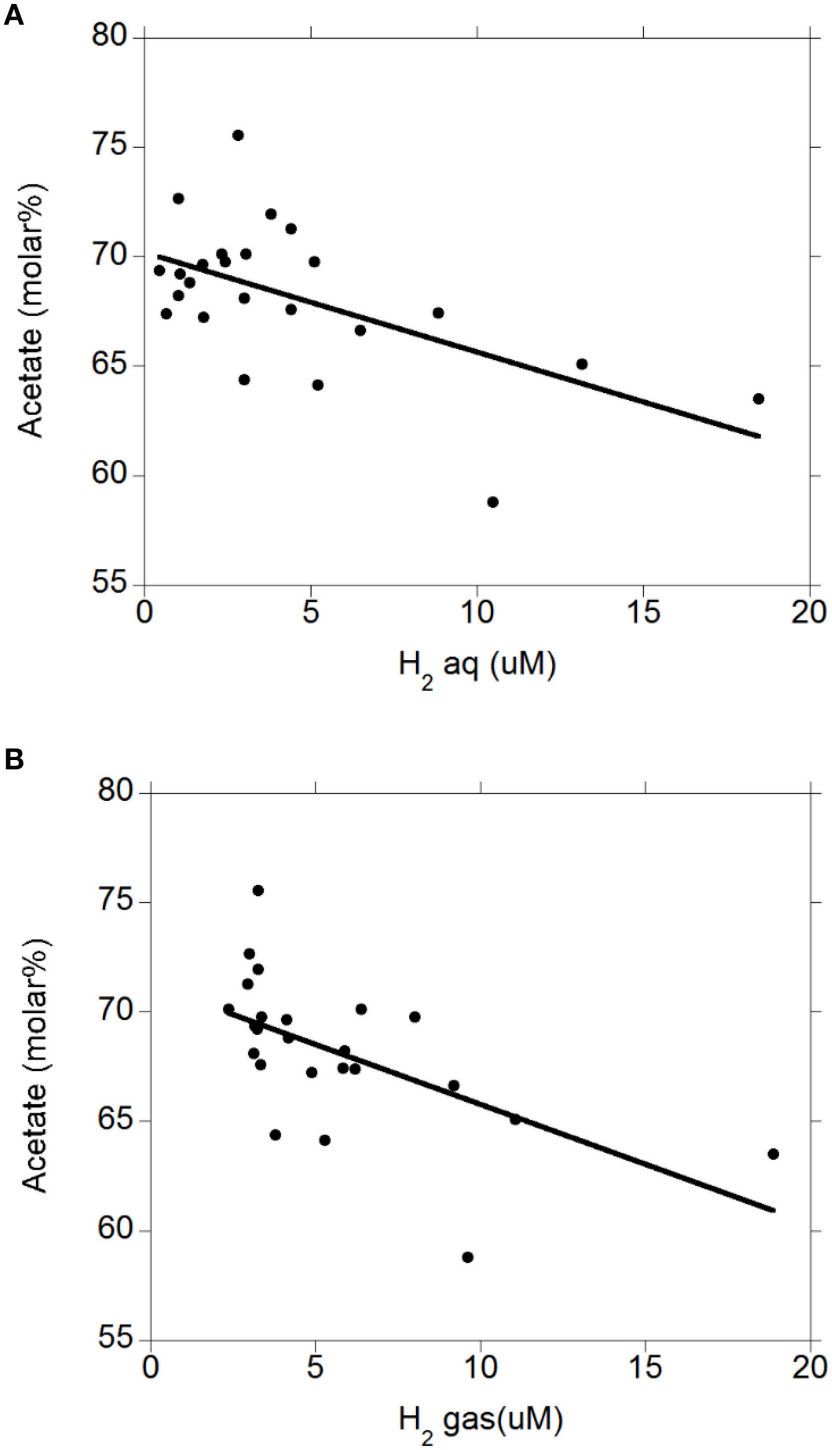
**Relationship between acetate molar percentage and concentration of dissolved and gaseous H_2_. (A)** acetate% = 70.2 (±0.84; *P* < 0.001) − 0.45 (±0.14; *P* = 0.003) H_2 (aq)_; *R*^2^ = 0.34 (*P* = 0.003); **(B)** acetate% = 71.3 (±1.07; *P* < 0.001) − 0.55 (±0.16; *P* = 0.003) H_2 (g)_; *R*^2^ = 0.36 (*P* = 0.003).

**Figure 5:**
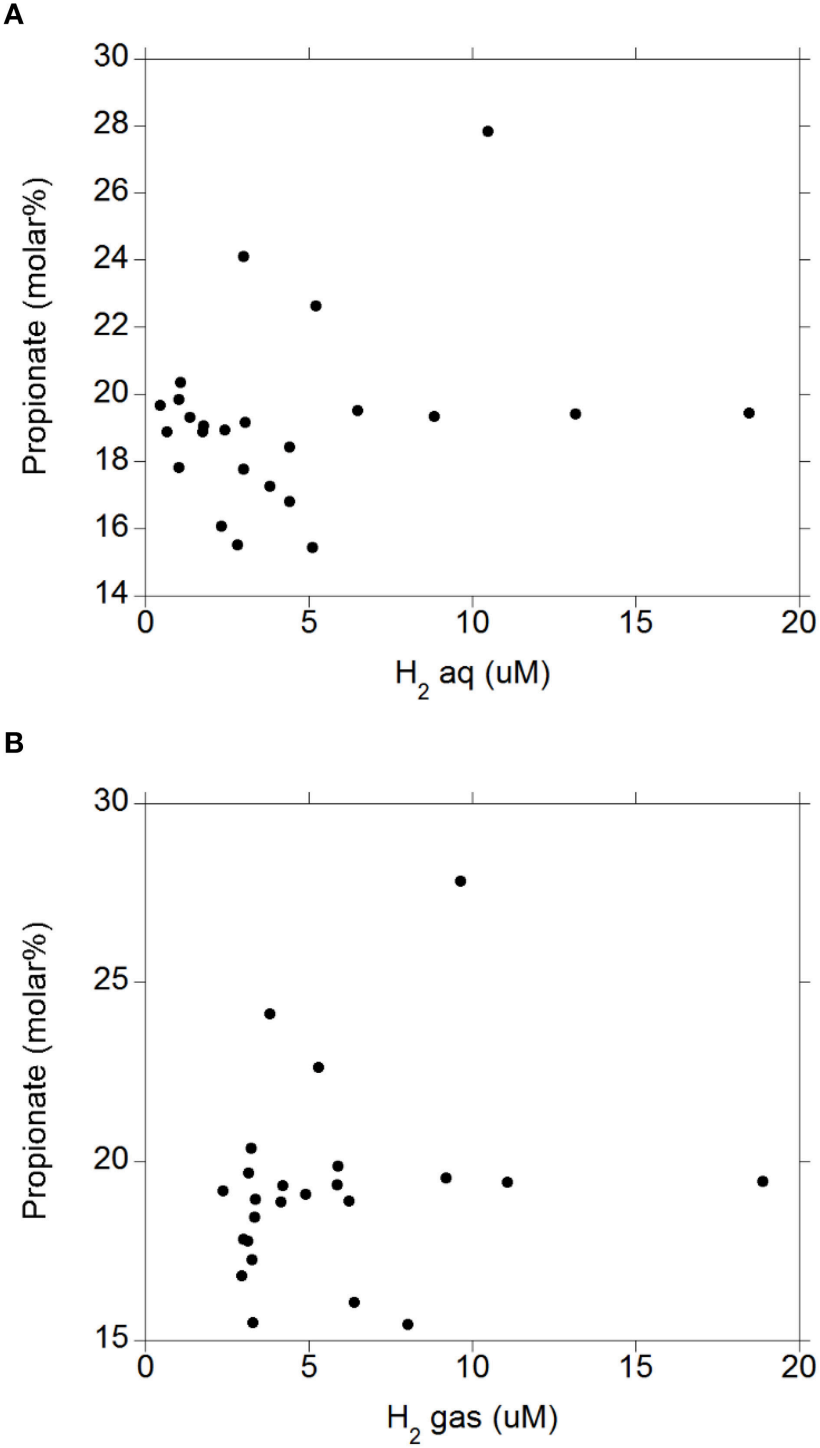
**Relationship between propionate molar percentage and concentration of dissolved and gaseous H_2_**. **(A)** propionate% = 18.6 (±0.78; *P* < 0.001) + 0.15 (±0.13; *P* = 0.24) H_2 (aq)_; *R*^2^ = 0.062 (*P* = 0.24); **(B)** H_2_: propionate% = 18.4 (±1.01; *P* < 0.001) + 0.14 (±0.15; *P* = 0.36) H_2 (g)_; *R*^2^ = 0.038 (*P* = 0.36).

**Figure 6:**
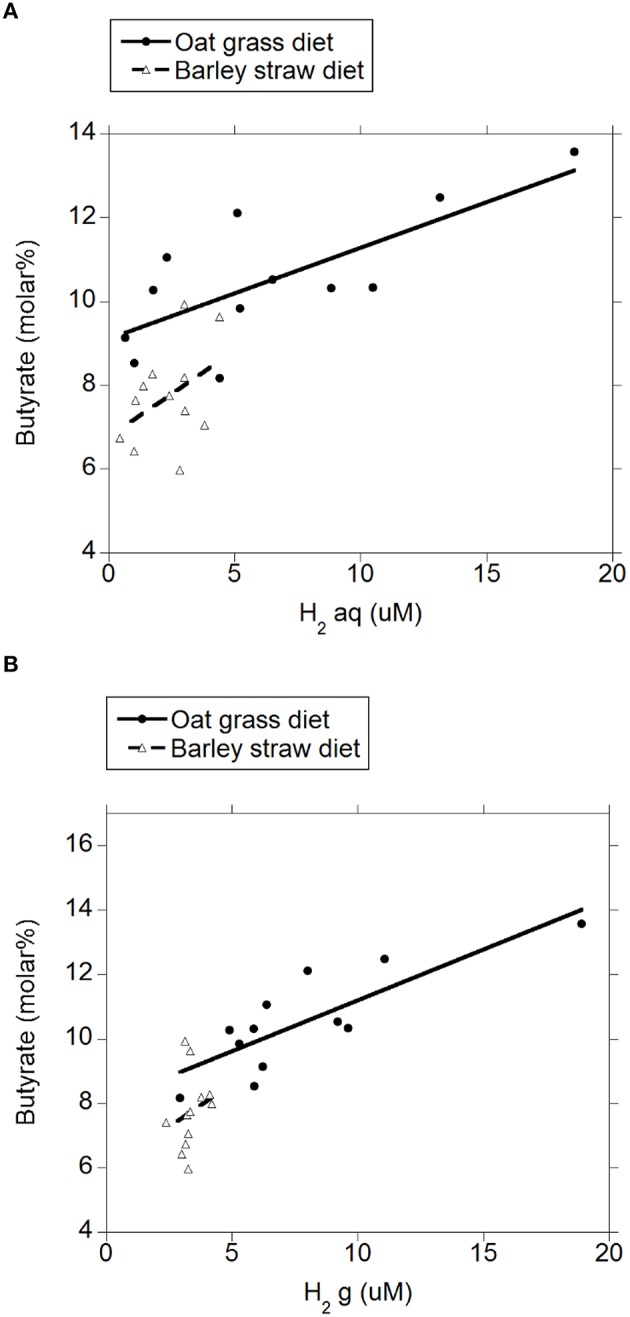
**Relationship between butyrate molar percentage and concentration of dissolved and gaseous H_2_. (A)** butyrate% = 8.14 (±0.35; *P* < 0.001) ± 0.92 (±0.26; *P* = 0.002) diet + 0.23 (±0.061; *P* = 0.001) H_2 (aq)_; *R*^2^ = 0.71 (*P* < 0.001); **(B)** butyrate% = 7.35 (±0.48; *P* < 0.001) ±0.67 (±0.28; *P* = 0.025) diet + 0.32 (±0.077; *P* < 0.001) H_2 (*g*)_; *R*^2^ = 0.66 (*P* < 0.001).

There were negative linear relationships between acetate molar percentage and H_2 (aq)_ (*P* = 0.003; Figure 4A) and H_2 (g)_ (*P* = 0.003; Figure 4B) without interactions with the diet (*P* > 0.71). There was no relationship between propionate molar percentage and H_2 (aq)_ (*P* = 0.24; Figure 5A) or H_2 (g)_ (*P* = 0.36; Figure 5B) concentration. There were positive linear relationships between butyrate molar percentage and H_2 (aq)_ (*P* = 0.001; Figure 6A) and H_2 (g)_ (*P* < 0.001; Figure 6B) concentrations.

There were no interactions between diet and method of Δ*G* estimation, so only main effects are presented (Table 4). With both diets, Δ*G* estimated using measured dissolved gases was greater (*P* < 0.001) for H_2_-producing reactions, and lesser for H_2_-incorporating reactions, when compared to Δ*G* estimated using dissolved gases concentration estimated from their gaseous phase concentration (Table 4).

**Table 4:** **Estimated Gibbs energy changes (kJ/reaction) of various rumen pathways**.

σ	**Diet**		**Method of Δ*G* estimation**		**SEM**	***P*****-value**		**Interaction**
	**Oat grass**	**Barley straw**	***Gas*_(*aq*)_**	***eGas*_(*aq*)_**		**Diet**	**Δ*G* estimation**	
Reaction 1	−340	−348	−326	−362	1.64	< 0.001	< 0.001	0.93
Reaction 2	−6.42	−0.59	−17.0	10.0	1.04	< 0.001	< 0.001	0.92
Reaction 3	9.79	13.6	2.69	20.7	0.776	< 0.001	< 0.001	0.93
Reaction 4	−340	−341	−341	−341	0.575	0.27	>0.999	>0.999
Reaction 5	−418	−413	−406	−424	1.25	0.004	< 0.001	0.95
Reaction 6	−335	−330	−323	−341	0.876	< 0.001	< 0.001	0.93
Reaction 7	−335	−342	−325	−352	1.26	< 0.001	< 0.001	0.93
Reaction 8	−5.18	2.10	−19.6	16.5	1.35	< 0.001	< 0.001	0.92
Reaction 9	−34.2	−26.6	−46.0	−14.8	1.88	< 0.001	< 0.001	0.95

*Reaction numbers correspond to sub-section 2.8 Calculation of the Gibbs energy changes of fermentation pathways: Reaction 1, glucose + 2 H_2_O → 2 acetate + 2 H^+^ + 2 CO_2_ + 4 H_2_; Reaction 2, acetate + CO_2_ + 3 H_2_ → propionate + 2 H_2_O; Reaction 3, 2 acetate + H^+^ + 2 H_2_ → butyrate + 2 H_2_O; Reaction 4, glucose → ⅔ acetate + 4/3 propionate + ⅔ CO_2_ + ⅔ H_2_O + 2 H^+^; Reaction 5, glucose → acetate + propionate + CO_2_ + H_2_ + 2H^+^; Reaction 6, glucose → butyrate + H^+^ + 2 CO_2_ + 2 H_2_; Reaction 7, glucose + H_2_O → acetate + ½ butyrate + 2 CO_2_ + 3 H_2_ + 3/2 H^+^; Reaction 8, 2 CO_2_ + 4 H_2_ → acetate + H^+^ + 2 H_2_O; Reaction 9, CO_2_ + 4 H_2_ → CH_4_ +2 H_2_O*.

*eGas _(aq)_, concentration of dissolved gases estimated from their concentration in the gas phase; Gas _(aq)_, concentration of dissolved gases directly measured; ΔG, Gibbs energy changes*.

## Discussion

### Effect of forage type on rumen fermentation end-products

Different forages influence rumen fermentation and CH_4_ production in ruminants, because dietary carbohydrate composition varies considerably with forage species and state of maturity (Chaves et al., 2006). Lower content of structural carbohydrates is associated with greater rate of digestion and fermentation, which in turn is associated with greater H_2_ concentration (Janssen, 2010). In our study, the OG diet had lower NDF and ADF content than the BS diet, which would agree with sheep consuming the OG diet having higher H_2 (aq)_ and CH_4 (aq)_ and total VFA concentration in the rumen compared to those fed the BS diet. Such differences seem likely to be due to the greater content of fermentable carbohydrates in the OG diet. In an *in vitro* experiment, higher H_2 (aq)_ and total VFA concentration were also observed for incubated substrates that typically have higher rate and extent of degradation, although rumen degradation was not measured in that study (Wang et al., 2014). Other factors potentially affected by the diet, such as rumen passage rate and fractional rate of VFA absorption, also influence the VFA concentration in the rumen (Dijkstra et al., 1993).

Sheep fed the OG diet had lower molar percentage of acetate and higher molar percentage of butyrate than sheep fed the BS diet. Higher NDF content in the BS diet may account for greater molar percentage of acetate (Brask et al., 2013). Rumen ammonia concentration was higher in the rumens of sheep fed BS diet, when compared with those fed the OG diet, even though the OG diet had more total N. Greater rumen ammonia concentration could suggest that incorporation of ammonia into carbon skeletons by rumen microbes was lower in sheep fed BS in comparison to the OG diet, perhaps as a result of lower supply of fermentable carbohydrates. Increased ammonia concentration and molar percentage of branched-chain VFA might also result from greater dietary and microbial protein degradation (Hassanat et al., 2014).

### Supersaturation of H_2_ and CH_4_ in the liquid phase of the rumen

In general, H_2 (aq)_ and H_2 (g)_ concentrations found in the present experiment are in the low end of H_2 (aq)_ and H_2 (g)_ concentrations ranges summarized by Janssen (2010). We attribute that in part to the low atmospheric pressure (0.64 atm) due to the elevation of the location where the experiment took place, as well as to the fact that sampling was conducted before the morning feeding, when H_2 (aq)_ concentration and H_2_ production are at their lowest point during the day (Robinson et al., 1981; Rooke et al., 2014).

Concentrations of H_2 (aq)_ and CH_4 (aq)_ were directly measured by establishing an equilibrium between gas and liquid phase in a sealed vessel (Wang et al., 2014). Concentrations of H_2 (aq)_ and CH_4 (aq)_ so determined were considerably greater than those estimated by assuming an equilibrium between the liquid and the gas phase of the rumen. Thus, assuming equilibrium for H_2_ or CH_4_ between the gas and liquid phases seems to be inappropriate to understand the rumen fermentation. The saturation factor was greatly larger than unity for H_2 (aq)_ and CH_4 (aq)_ in the rumen, indicating that both H_2_ and CH_4_ were supersaturated in rumen fluid. Furthermore, H_2_ supersaturation might have been even greater a few hours after feed was offered, when H_2 (aq)_ concentration is greatest (Robinson et al., 1981), as there was a positive relationship between the saturation factor and gases concentrations (Figure 3). The supersaturation of H_2_ and CH_4_ indicates mass-transfer limitations to the movement of both H_2_ and CH_4_ from the liquid to gaseous phase in the rumen. Therefore, it does not seem appropriate to use H_2 (g)_ concentration to predict rumen H_2 (aq)_ concentration, which is the variable important to H_2_-producing and H_2_-utilizing microorganisms. Likewise, CH_4 (aq)_ concentration directly measured in the fluid appears as a more reliable indicator of methanogens activity than CH_4 (g)_ concentration.

Inhibiting methanogenesis in rumen *in vitro* batch and continuous mixed cultures consistently decreased the recovery of metabolic hydrogen in propionate, butyrate, CH_4_ and H_2_ (Ungerfeld, 2015b). In that analysis, metabolic hydrogen in H_2_ was calculated taking into account only published data on H_2 (g)_, but H_2 (aq)_ was not reported in the studies used for the analysis by Ungerfeld (2015b) and was thus not considered. We now estimated H_2 (aq)_ for the experiments used for the analysis by Ungerfeld (2015b) using the reported concentrations of H_2 (g)_ based on the relationship between H_2 (aq)_ and H_2 (g)_ found in the present study (Figure 1). Adding reducing equivalents in H_2 (aq)_ to the calculation of metabolic H_2_ recovery of the study by Ungerfeld (2015b) resulted in a marginal increase in the recovery of metabolic H_2_predicted for 100% methanogenesis inhibition, of between 1 and 2% in batch culture and about 1% in continuous culture (calculations not shown). Reducing equivalents in estimated CH_4 (aq)_ were not calculated and added because in the present experiment CH_4 (aq)_ was unrelated to CH_4 (g)_, and therefore adding reducing equivalents in CH_4 (aq)_ would only affect the intercept of the relationship between metabolic hydrogen recovery and the inhibition of methanogenesis but not the slope of their relationship.

### Association of dissolved and gaseous H_2_ with rumen fermentation pathways

Rumen H_2_ concentration affects H_2_-producing and H_2_-incorporating pathways in the rumen (Ellis et al., 2008), with lower H_2 (aq)_ concentration favoring acetate production, whereas greater H_2 (aq)_ concentration favors propionate and butyrate production (Janssen, 2010; Wang et al., 2014). In agreement, we observed that both measured and estimated H_2 (aq)_ concentration were negatively correlated with the molar percentage of acetate, and positively and linearly correlated with the molar percentage of butyrate, although there was no relationship with the molar percentage of propionate. Methanogens, as major H_2_-utilizing microorganisms in the rumen, have a Monod relationship of growth with H_2 (aq)_ concentration, with the *Ks*-values (i.e., half the maximum growth rate) ranging from 4 to 9 uM of H_2 (aq)_ concentration (Janssen, 2010). As expected, we observed increased CH_4 (aq)_ concentration with greater H_2 (aq)_ concentration.

Gibbs energy changes of glucose fermentation in pathways producing H_2_ (Reactions 1, 5–7) were greater (i.e., less favorable) using measured H_2 (aq)_ compared with eH_2 (aq)_ estimated from H_2 (g)_ concentration by assuming equilibrium. On the other hand, Δ*G* of H_2_-incorporating reactions such as acetate conversion to propionate (Reactions 2) and to butyrate (Reactions 3), reductive acetogenesis (Reactions 8), and methanogenesis (Reactions 9), were lower using measured H_2 (aq)_ concentration compared to eH_2 (aq)_ estimated from H_2 (g)_. Furthermore, the thermodynamic ranking of glucose fermentation pathways changed when calculations were made using measured dissolved gases concentrations instead of dissolved gases concentrations estimated by assuming equilibrium with their corresponding concentrations in the gas phase. For example, Reaction 1 was the second to most favorable H_2_-releasing pathway when using directly measured dissolved gases, but it was among the least favorable H_2_-releasing pathway if using dissolved gases concentrations estimated from their corresponding concentrations in the rumen gaseous phase. It can be concluded that Δ*G* differed when calculations were made using measured instead of estimated dissolved gases concentration.

## Conclusions

Measured H_2 (aq)_ and CH_4 (aq)_ were greater than H_2 (aq)_ and CH_4 (aq)_ concentrations estimated by assuming equilibrium with the gas phase, indicating that both H_2_ and CH_4_ were supersaturated in liquid phase of rumen. Thus, H_2 (aq)_ or CH_4 (aq)_ concentration estimated by assuming equilibrium with the gaseous phase do not seem appropriate for calculating Δ*G* of reactions that involve release of H_2_ or CH_4_, or H_2_ incorporation, in the liquid phase of rumen. Concentration of H_2 (aq)_ was positively correlated with CH_4 (aq)_ concentration and the molar percentage of butyrate, and negatively correlated with molar percentage of acetate, confirming that changes in H_2 (aq)_ concentration are associated with shifts of rumen fermentation pathways and the extent of CH_4_ generation. To our knowledge, this is the first *in vivo* study in which fluid and gaseous phase concentration of H_2_ and CH_4_ in the rumen were related. These relationships need to be studied within a much wider range of conditions, including different animals, diets, and changes throughout the day, as well as locations situated at lower elevation, among other factors. The relationships of H_2 (aq)_ and CH_4 (aq)_ with microbial populations are also of much interest.

## Author contributions

MW, ZT, and CZ designed research; MW, ZB, SA, and RW conducted research; MW and EU analyzed data; MW, ZT, and EU wrote the paper. All authors read and approved the final manuscript.

### Conflict of interest statement

The authors declare that the research was conducted in the absence of any commercial or financial relationships that could be construed as a potential conflict of interest.
